# Deoxyshikonin: a promising lead drug grass against drug resistance or sensitivity to *Helicobacter pylori* in an acidic environment

**DOI:** 10.1128/aac.00959-24

**Published:** 2024-08-22

**Authors:** Jia-yin Xu, Hui-hua Dong, Li-juan Liao, Shi-xian Yang, Lu-yao Wang, Hao Chen, Peipei Luo, Liang Huang, Ai-xing Guan, Yan-Qiang Huang

**Affiliations:** 1 Guangxi Technology Innovation Cooperation Base of Prevention and Control Pathogenic Microbes with Drug Resistance, Youjiang Medical University for Nationalities, Baise, China; 2 Affiliated Hospital of Youjiang Medical University for Nationalities, Baise, China; 3 Guangxi Zhuang Autonomous Region Engineering Research Center of Clinical Prevention and Control Technology and Leading Drug for Microorganisms with Drug Resistance in Border Ethnic Areas, Baise, China; 4 Department of Pathology, School of Basic Medical Sciences, Wannan Medical College, Wuhu, China; 5 Department of Gastroenterology, Wujin People’s Hospital affiliated to JiangSu University, Changzhou, China; 6 Guangxi Clinical Medical Research Center for Hepatobiliary Diseases, Liuzhou, China; Columbia University Irving Medical Center, New York, New York, USA

**Keywords:** *Helicobacter pylori*, deoxyshikonin, inhibitory action, lead drug, acidic environment

## Abstract

*Helicobacter pylori* (*H. pylori*) is closely associated with the diseases such as gastric sinusitis, peptic ulcers, and gastric adenocarcinoma. Its drug resistance is very severe, and new antibiotics are urgently needed. Nine comfrey compounds were screened by antimicrobial susceptibility testing, among which deoxyshikonin had the best inhibitory effect, with a minimum inhibitory concentration (MIC) of 0.5–1 µg/mL. In addition, deoxyshikonin also has a good antibacterial effect in an acidic environment, it is highly safe, and *H. pylori* does not readily develop drug resistance. Through *in vivo* experiments, it was proven that deoxyshikonin (7 mg/kg) had a beneficial therapeutic effect on acute gastritis in mice infected with the multidrug-resistant *H. pylori* BS001 strain. After treatment with desoxyshikonin, colonization of *H. pylori* in the gastric mucosa of mice was significantly reduced, gastric mucosal damage was repaired, inflammatory factors were reduced, and the treatment effect was better than that of standard triple therapy. Therefore, deoxyshikonin is a promising lead drug to solve the difficulty of drug resistance in *H. pylori*, and its antibacterial mechanism may be to destroy the biofilm and cause an oxidation reaction.

## INTRODUCTION

*Helicobacter pylori* is a Gram-negative, spiral bacterium that causes a variety of gastrointestinal diseases such as gastritis, chronic gastritis, peptic ulcers, and gastric adenocarcinoma (1). The prevalence of *H. pylori* infection varies by age, ethnicity, and diet (2). The infection rate of *H. pylori* among young Chinese individuals aged 20–40 years is 40%–70%, accounting for half of the total infected population, with those aged over 70 years being at high risk of infection (3). The current treatment options for *H. pylori* infections include standard triple therapy or bismuth-containing quadruple therapy (two antibiotics). Long-term antibiotic use leads to increased antibiotic resistance in *H. pylori* and reduced eradication rates (4–7). Therefore, for the health of the global population, there is an urgent need to develop new drugs to eradicate drug-resistant *H. pylori*.

*Radix lithospermi* is a herbal medicine included in the Chinese pharmacopoeia made with the dried roots of *Radix lithospermi* or *Arnebia gutlata* Bunge, all from the comfrey plant as used for medicinal purposes. It promotes lymphangiogenesis and wound healing and has antibacterial and anticancer effects and other pharmacological actions (8, 9). Deoxyshikonin as a chemical component of *Radix lithospermi* inhibits drug resistance of non-small cells causing lung carcinoma to cisplatin by suppressing the ABCB1 expression in Akt signaling (10). In addition, deoxyshikonin exerted a better inhibitory effect on the formation of *Staphylococcus aureus* biofilms than on that of *Escherichia coli* and *Pseudomonas aeruginosa* (11). Vukic *et al*. performed antibacterial tests using components of *Radix lithospermi* on five Gram-positive bacteria (*Bacillus megaterium, Enterococcus faecalis*, *Microbacterium arborescens*, *micrococcus luteus*, *and Staphylococcus epidermidis*) and five Gram-negative bacteria (*Citrobacter koseri*, *Hafnia alvei*, proteolytic *Pseudomonas* strains, *Stenotrophomonas maltophilia*, and *Yersinia intermedia*) (12). α-methylbutyrylshikonin and acetylshikonin demonstrated good bacteriostatic activity against the aforementioned Gram-positive and Gram-negative bacteria (13–16). However, no reports on the inhibitory effects of *Radix lithospermi* and deoxyshikonin on *H. pylori* are available. Since *H. pylori* is a special bacterium that grows in the acidic environment, many drugs do not work well in the acidic environment, so it is necessary to study the antimicrobial effect of deoxyshikonin on *H. pylori* therein.

## RESULTS

### The relationship between chemical structure and antibacterial effect

**Fig 1:**
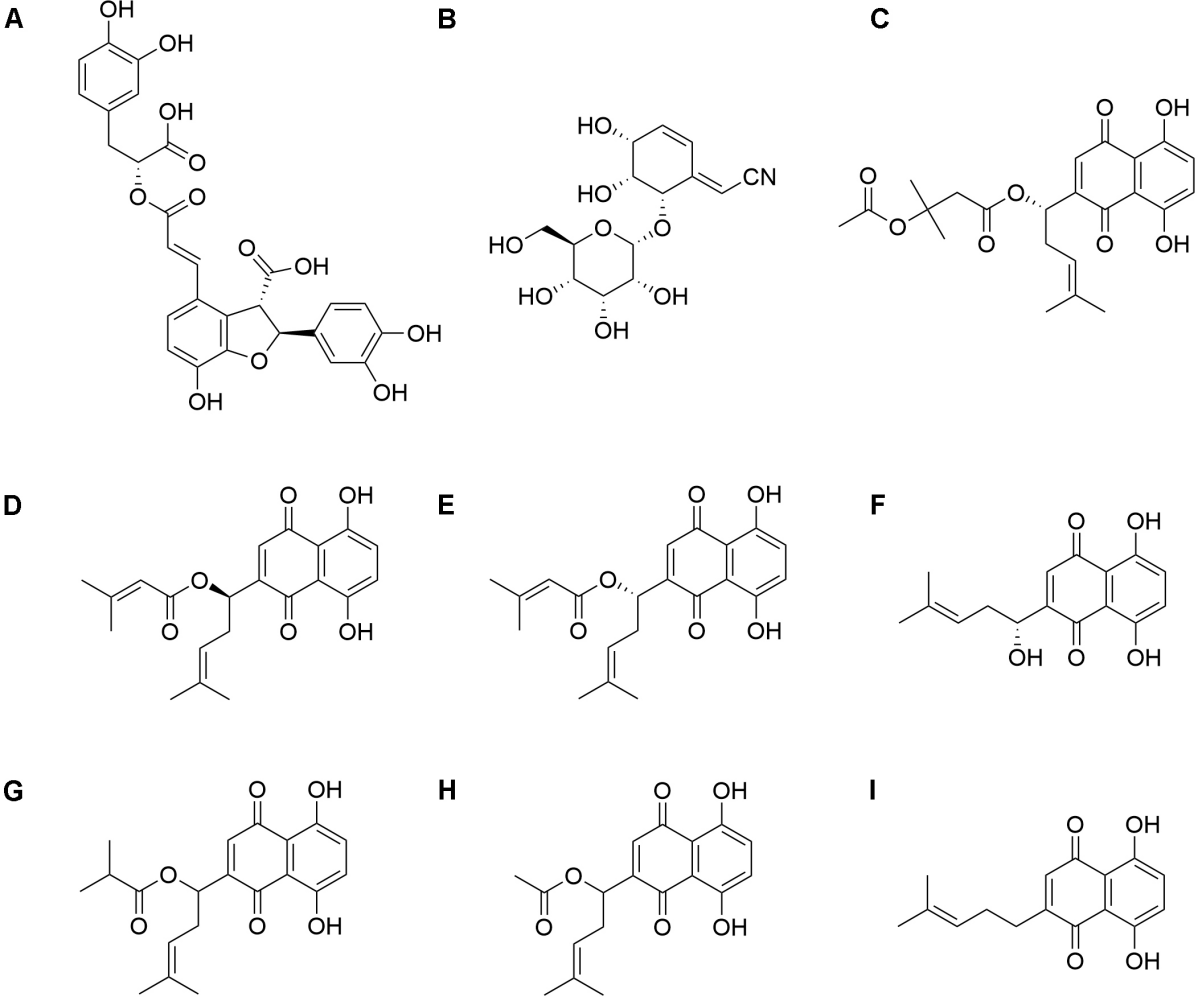
Chemical structure of nine components of *Radix lithospermi*. (A) Lithospermic acid; (B) cyanoside; (C) β-acetoxyisovaleryl akanin; (D) β,β-dimethylacryloyl shikonin; (E) β,β-dimethylacryloyl akanin; (F) shikonin; (G) isobutyryl shikonin; (H) acetylshikonin; (I) deoxyshikonin.

Different functional groups and their different positions can lead to differences in antibacterial activity (Fig. 1). Lithospermic acid contains two parts: phenylpropanoid and shikonin. β-acetoxyisovaleryl akanin contains multiple different functional groups such as acetoxy and isoprene groups. β, β-dimethylacryloyl akanin contains a dimethylacryloyl functional group. Shikonin has a complex multiring structure. These complex structures may complicate its metabolic pathways *in vivo*, reduce its solubility in aqueous solutions, limit its absorption and distribution *in vivo*, reduce its bioavailability and antibacterial activity, and may also affect its binding mode or affinity with target molecules.

The isobutyryl functional group in isobutyryl shikonin replaces other functional groups in shikonin, which may alter the electron density and spatial configuration of the molecule. Acetylshikonin has an acetyl functional group substitution. The presence of cyanide groups in cyanoside may lead to unstable reactions of the compound under certain conditions. The structure of β, β-dimethylacryloyl shikonin contains many double bonds, which may cause it to undergo photo-oxidation or other unstable reactions under certain conditions, leading to decomposition or deactivation. These instabilities may reduce the persistence and efficacy of their antibacterial activity. Due to the lack of an oxygen atom in deoxyshikonin, its structure may be more compact and stable, increasing its binding efficiency with target molecules in organisms and reducing its susceptibility to degradation by metabolic enzymes. This may be beneficial for enhancing its antibacterial activity *in vivo*.

### Screening of antibacterial active components of agrimonin *in vitro*

The antibacterial activities of nine components found in *Radix lithospermi* were detected using the broth dilution method, including shikosin, lithospermoside, β,β-dimethylacryl shikonin, β,β-dimethyl-acryl-alkannin, lithospermic acid, β-acetoxyisovalerylalkannin, isobutyrylshikonin, acetylshikonin, and deoxyshikonin. The results indicate that deoxyshikonin exerted best antibacterial effects on *H. pylori* among them (MIC_90_, 0.5 µg/mL); however, it was found that deoxyshikonin had no inhibitory effects on *Klebsiella pneumoniae*, *P. aeruginosa*, *Acinetobacter baumannii*, *S. aureus*, and other bacteria, with all MIC_90_ values exceeding 128 µg/mL (Table 1). The present study clarifies that the main component of lithospermum, which inhibits *H. pylori*, is deoxyshikonin and thus the focus of subsequent research.

**TABLE 1:** MIC of related components of *Radix lithospermi* (μg/mL)

Component	HPBS001	Hp26695	*Klebsiella* *pneumoniae*	*Pseudomonas* *aeruginosa*	*Acinetobacter* *baumannii*	*Staphylococcus* *aureus*
Shikonin	2	2	> 128	> 128	> 128	> 128
Lithosprmoside	> 128	> 128	> 128	> 128	> 128	> 128
β,β-Dimethylacrylshikonin	32	32	> 128	> 128	> 128	> 128
β,β-Dimethylacrylalkannin	32	32	> 128	> 128	> 128	> 128
Lithospermic acid	> 128	128	> 128	> 128	> 128	> 128
β-Acetoxyisovalerylshikonin	8	16	> 128	> 128	> 128	> 128
Isobutyrylshikonin	8	8	> 128	> 128	> 128	> 128
Acetylshikonin	4	2	> 128	> 128	> 128	> 128
Deoxyshikonin	1	0.5	> 128	> 128	> 128	> 128

**Fig 2:**
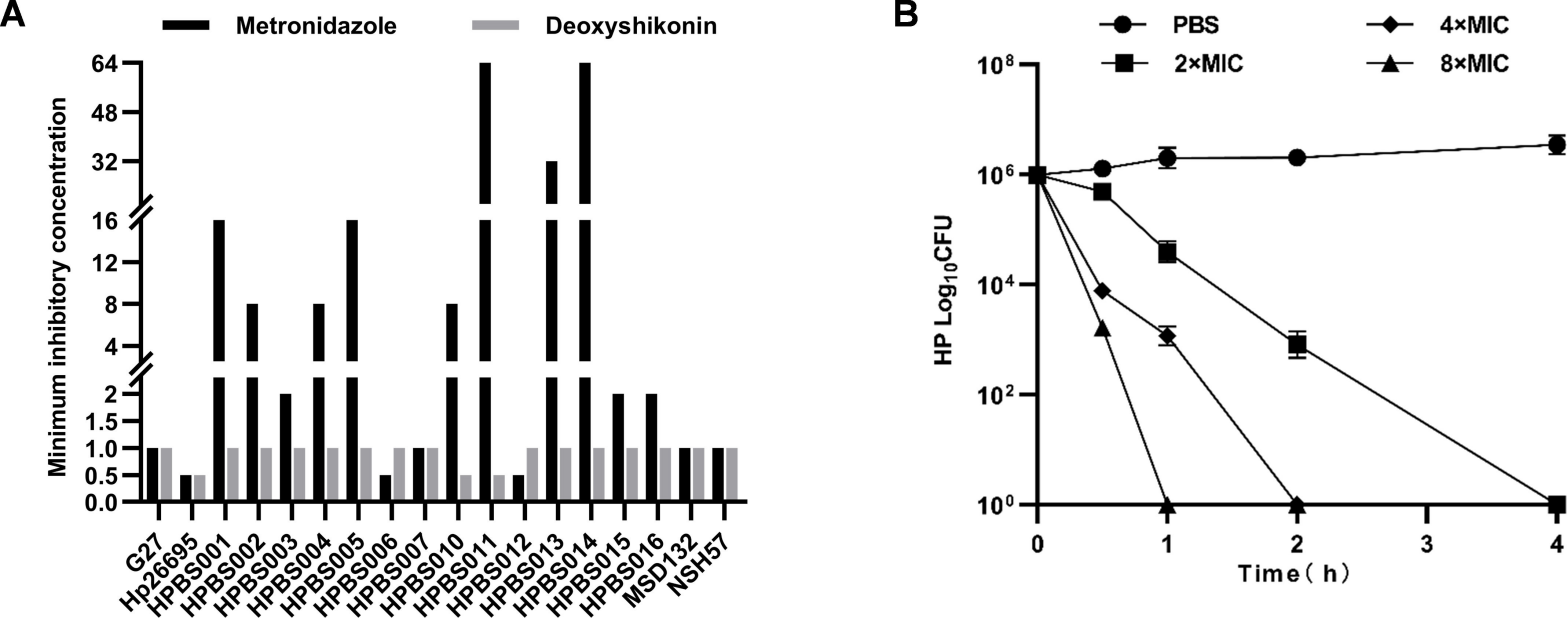
Antimicrobial activity of deoxyshikonin. (A) The minimum inhibitory concentration (MIC) of deoxyshikonin; (B) the minimum bactericidal concentration (MBC) of deoxyshikonin.

The antibacterial effects of deoxyshikonin on 18 *H*. *pylori* strains from different sources were detected using a microdilution method. It was found that deoxyshikonin had good inhibitory effects on all *H. pylori* strains, both seven sensitive strains and eleven resistant strains, with an MIC of 0.5–1 µg/mL (Table 2), and the minimum bacterial concentration (MBC) 99.9% was 2 µg/mL at 2 hours (Fig. 2).

**TABLE 2:** MIC of metronidazole and deoxyshikonin against the different *H. pylori* strains

Strains	Drug resistance	MIC (μg/mL)	
		Metronidazole	Deoxyshikonin
*G27*	Sensitive	1	1
*Hp26695*	Sensitive	0.5	0.5
*HPBS001*	Resistant to LEV, CLA, and MET	16	1
*HPBS002*	Resistant to MET	8	1
*HPBS003*	Resistant to CLA	2	1
*HPBS004*	Resistant to LEV	8	1
*HPBS005*	Resistant to LEV and MET	16	1
*HPBS006*	Resistant to CLA	0.5	1
*HPBS007*	Resistant to CLA	1	1
*HPBS010*	Resistant to LEV, CLA, and MET	8	0.5
*HPBS011*	Resistant to CLA and MET	64	0.5
*HPBS012*	Sensitive	0.5	1
*HPBS013*	Resistant to LEV, CLA, and MET	32	1
*HPBS014*	Resistant to LEV, CLA, AMO, and MET	64	1
*HPBS015*	Sensitive	2	1
*HPBS016*	Sensitive	2	1
*MSD132*	Sensitive	1	1
*NSH57*	Sensitive	1	1

### Safety of deoxyshikonin *in vivo*

**Fig 3:**
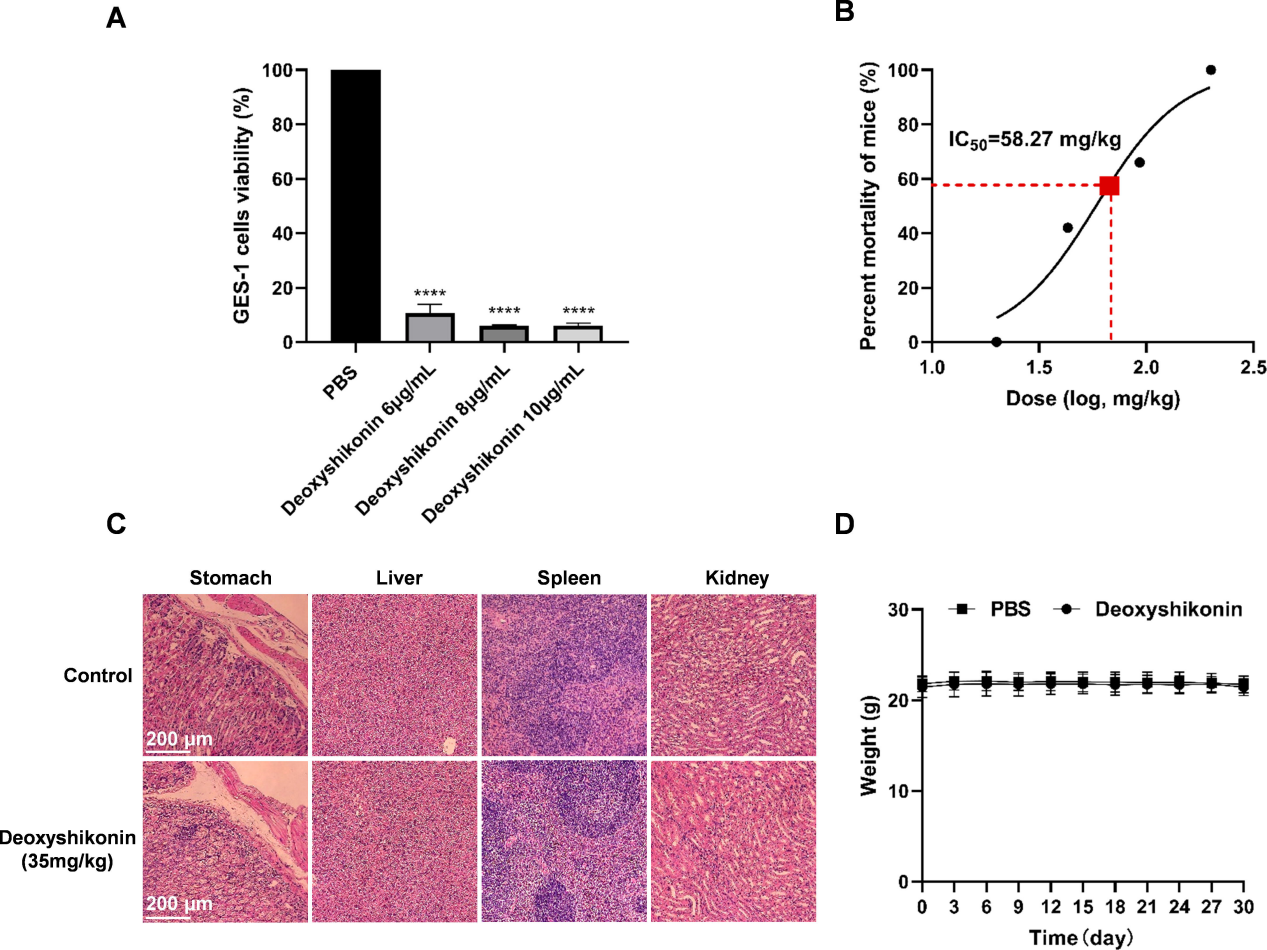
Safety of deoxyshikonin *in vivo*. (A) Cytotoxicity of deoxyshikonin to GES-1 cells; (B) dosage response mortality curves of deoxyshikonin; (C) effects of deoxyshikonin on the organs of mice; (D) effects of deoxyshikonin on the weights of mice.

*In vitro* cytotoxicity experiments showed that deoxyshikonin has certain toxicity to normal gastric epithelial cells GES-1 (Fig. 3A). The LD_50_ of deoxyshikonin determined by intraperitoneal injection was 58.27 mg/kg, indicating low toxicity and high safety of deoxyshikonin in mice (Fig. 3B). The safety test was conducted via intragastric administration of five times the therapeutic dose of deoxyshikonin (35 mg/kg) to SPF C57BL/6 mice, and no evident pathological damage occurred in the stomach, liver, spleen, and kidneys (Fig. 3C). The mice were weighed for 30 days, consecutively, and there was no significant change in their weights (Fig. 3D).

### Antibacterial activity of deoxyshikonin *in vivo*

**Fig 4:**
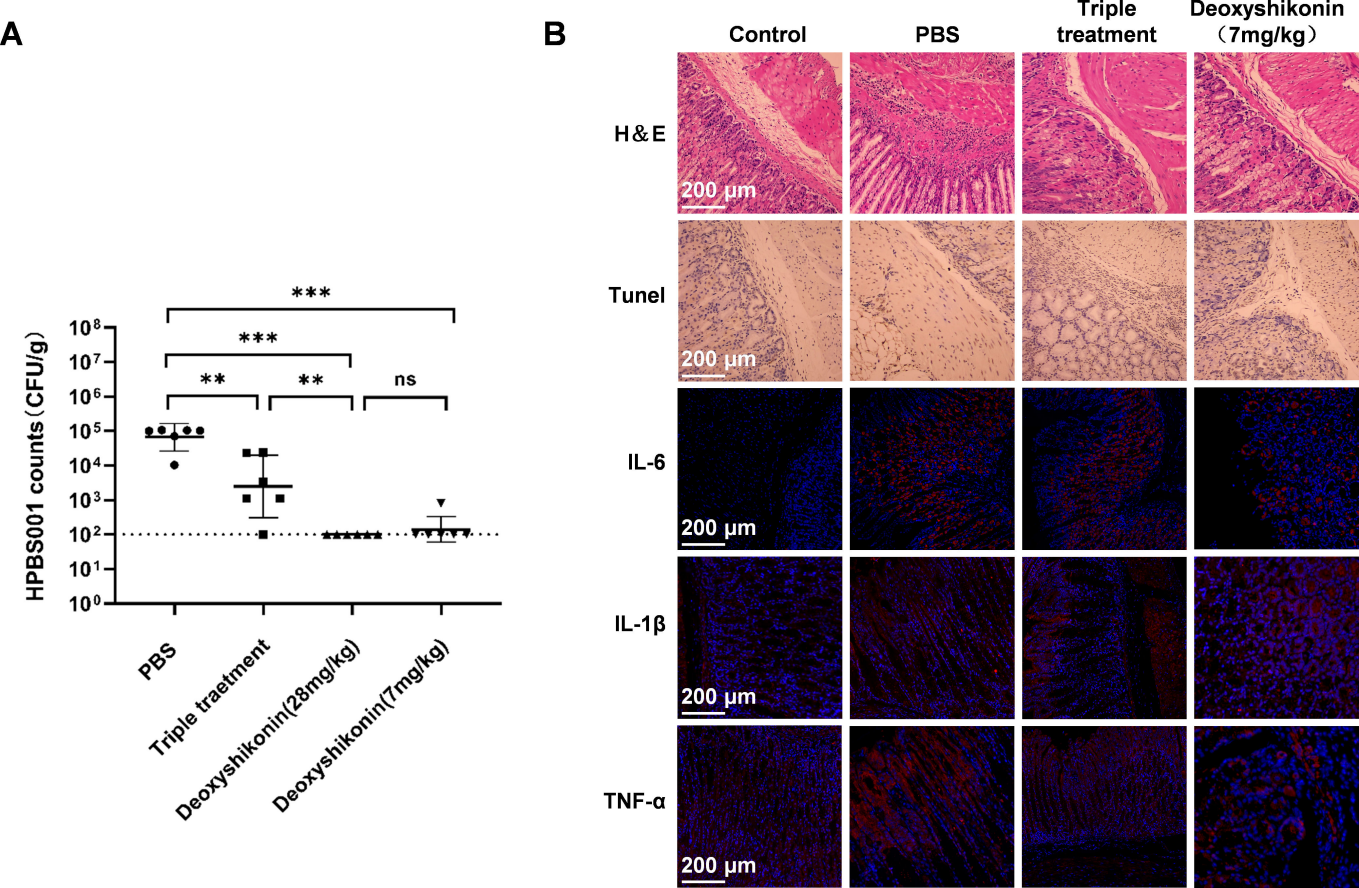
Antibacterial activity of deoxyshikonin *in vivo*. (A) Colonization of *H. pylori* in mice with acute gastritis after treatment with deoxyshikonin; (B) repair of gastric mucosa of mice with acute gastritis after treatment with deoxyshikonin (10×).

The clinical *H. pylori* BS001 strain was induced by adaptive colonization and inoculated into C57BL/6 mice to establish model mice with acute gastritis following the process as previously described.The antibacterial activity *in vivo* of deoxyshikonin was evaluated. It was found that the deoxyshikonin group could produce therapeutic effects significantly better than the triple therapy group. However, a dosage of 7 mg/kg could almost inhibit all *H. pylori* adhesion and colonization in the stomach (Fig. 4A). HE staining and TUNEL staining were performed on tissues of gastric mucosae. The results showed few apoptotic and inflammatory cells and light tissue damage in the deoxyshikonin treatment group, with no significant difference compared with the normal group (Fig. 4B). The expressions of IL-6, TNF-α, and IL-1β detected through immunofluorescence staining were all significantly reduced (Fig. 4B), indicating that deoxyshikonin inhibited the adhesion and colonization of *H. pylori* in the gastric mucosa of mice, reduced the inflammatory response, and repaired the damage thereto.

### Deoxyshikonin inhibits flagellar movement of *H. pylori* and enhances its membrane permeability and oxidation

**Fig 5:**
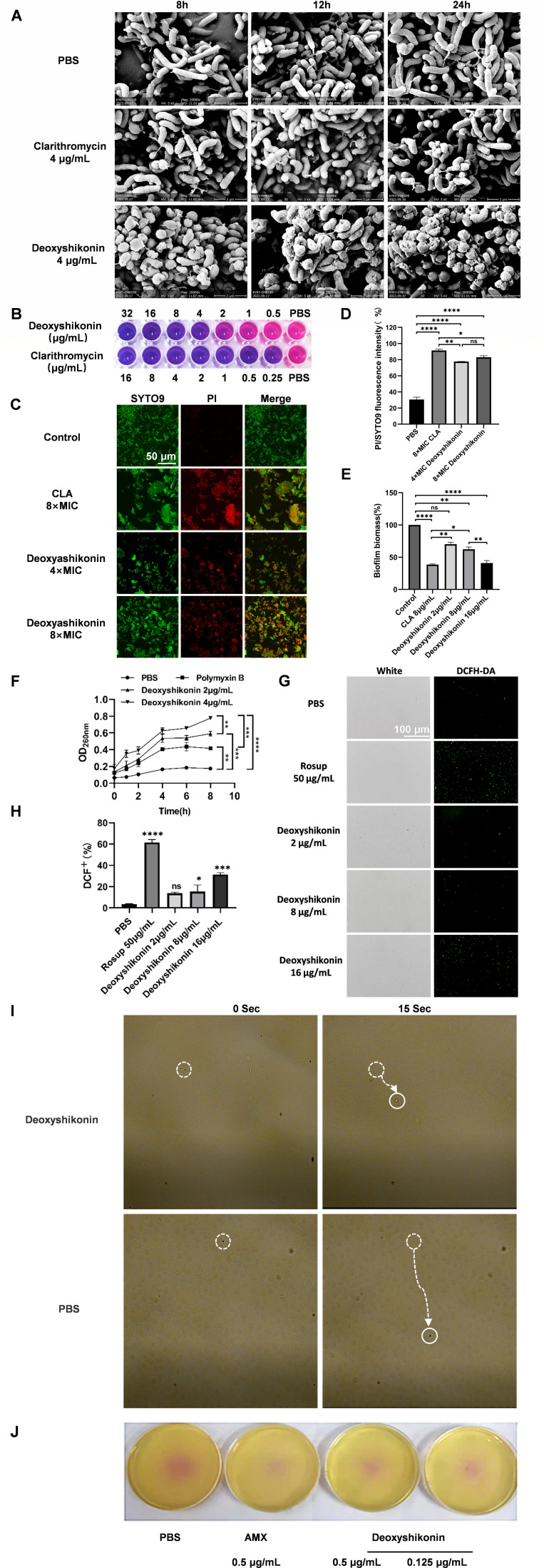
Deoxyshikonin inhibits the growth of *H. pylori* biofilms and movement of flagella and enhances the permeability of cell membranes as well as oxidation. (A) Morphological and structural damage caused by *H. pylori*; (B) the effect of deoxyshikonin on *H. pylori* biofilm detected using Alamar staining; (C) the effect of deoxyshikonin on the *H. pylori* biofilm detected using a confocal microscope after staining with SYTO9; (D) the effect of deoxyshikonin on the *H. pylori* biofilm detected quantitatively after I/SYTO9 staining; (E) the effect of deoxyshikonin on the *H. pylori* biofilm detected through crystal violet staining; (F) the osmotic damage of the membrane of *H. pylori* cells caused by deoxyshikonin; (G) the oxidation effect of deoxyshikonin on *H. pylori* as detected quantitatively; (H) the oxidation effect of deoxyshikonin on *H. pylori* detected using fluorescence staining; (I) the suspension drop method to observe the inhibitory effect of deoxyshikonin on the movement of *H. pylori* (40 x); (J) detection of the inhibitory effect of deoxyshikonin on *H.pylori* flagella by soft agar assay.

In the present study, the effects of deoxyshikonin on the morphology and structure of *H. pylori* were detected using an SEM. After treatment with four times the MIC of deoxyshikonin on *H. pylori* for 24 hours, spherical changes and roughness were detected on the cell surface (Fig. 5A). When detecting the permeability of the *H. pylori* membrane, it was found that deoxyshikonin destroyed the biofilm (Fig. 5B through E) and enhanced the permeability of the cell membrane in a concentration-dependent manner, and a dose of 2 µg/mL could exert better effects on enhancing cell membrane permeability than did polymyxin B (Fig. 5F). When detecting the oxidation of deoxyshikonin on *H. pylori*, it was found that the oxidation reaction occurred in a concentration-dependent manner and became stronger at 16 µg/mL (Fig. 5G and H). In soft agar and exercise experiments, deoxyshikonin can effectively inhibit the movement of *H. pylori* flagella (Fig. 5I and J).

### Advantages and characteristics

**Fig 6:**
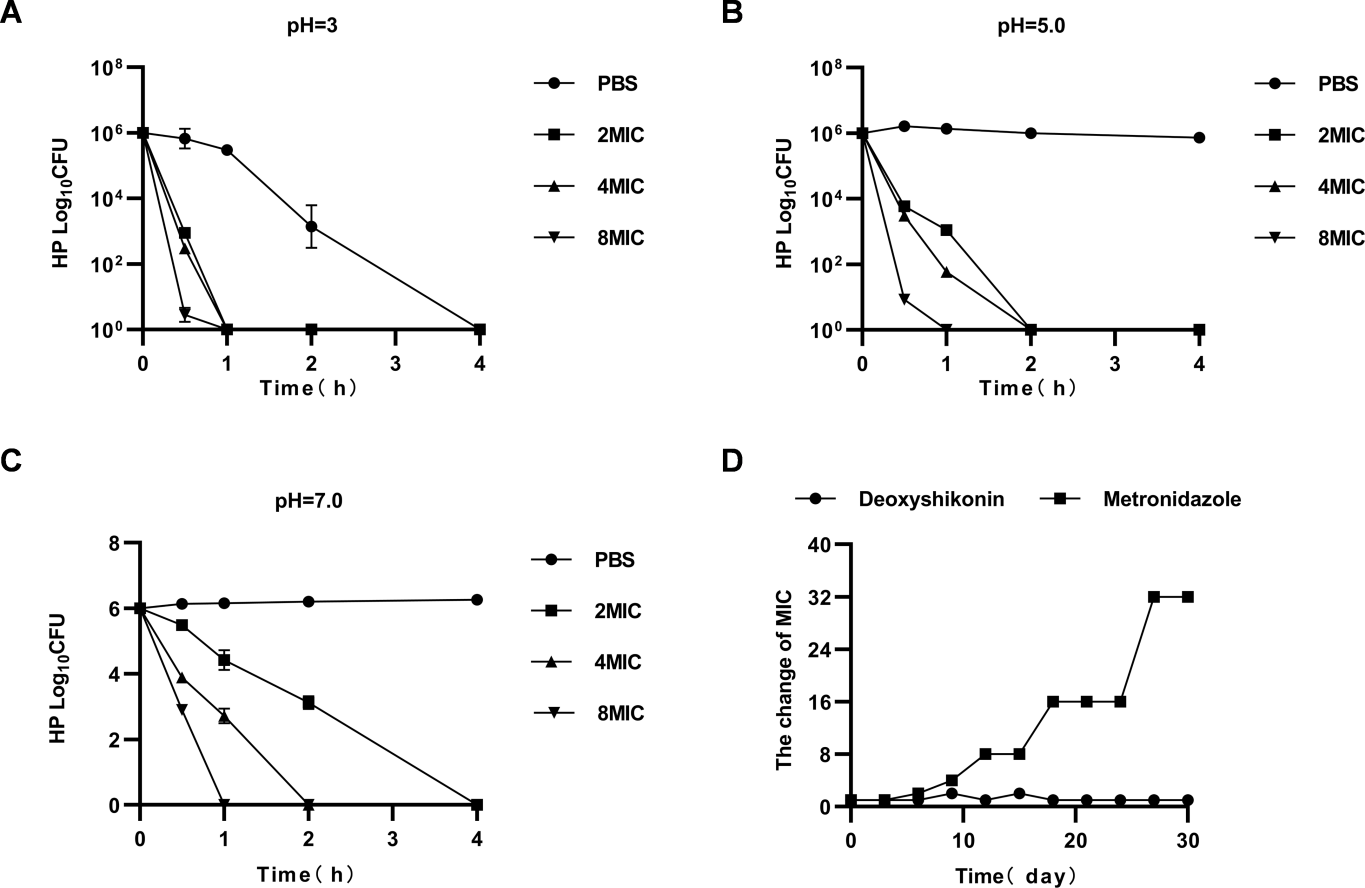
Acid resistance testing for deoxyshikonin and detection of *H. pylori* resistance. (A) MBC of deoxyshikonin at pH = 3.0; (B) MBC of deoxyshikonin at pH = 5.0; (C) MBC of deoxyshikonin at pH = 7.0; (D) detection of drug resistance of *H. pylori* to deoxyshikonin and metronidazole.

After deoxyshikonin was co-incubated with *H. pylori* in the culture medium at pH 3.0, pH 5.0, and pH 7.0 for 1 hour, its inhibitory effects were assessed. The results suggest that the number of viable bacteria of pH 3.0 and 5.0 in the PBS group were significantly decreased over time, especially at pH 3.0 when the number of viable bacteria was less than 10, whereas no significant change was observed in the PBS group (Fig. 6A through C). This finding indicates that deoxyshikonin demonstrated better antibacterial effects at a faster rate in an acidic environment. *H. pylori* was not easily resistant to deoxyshikonin (Fig. 6D).

**Fig 7:**
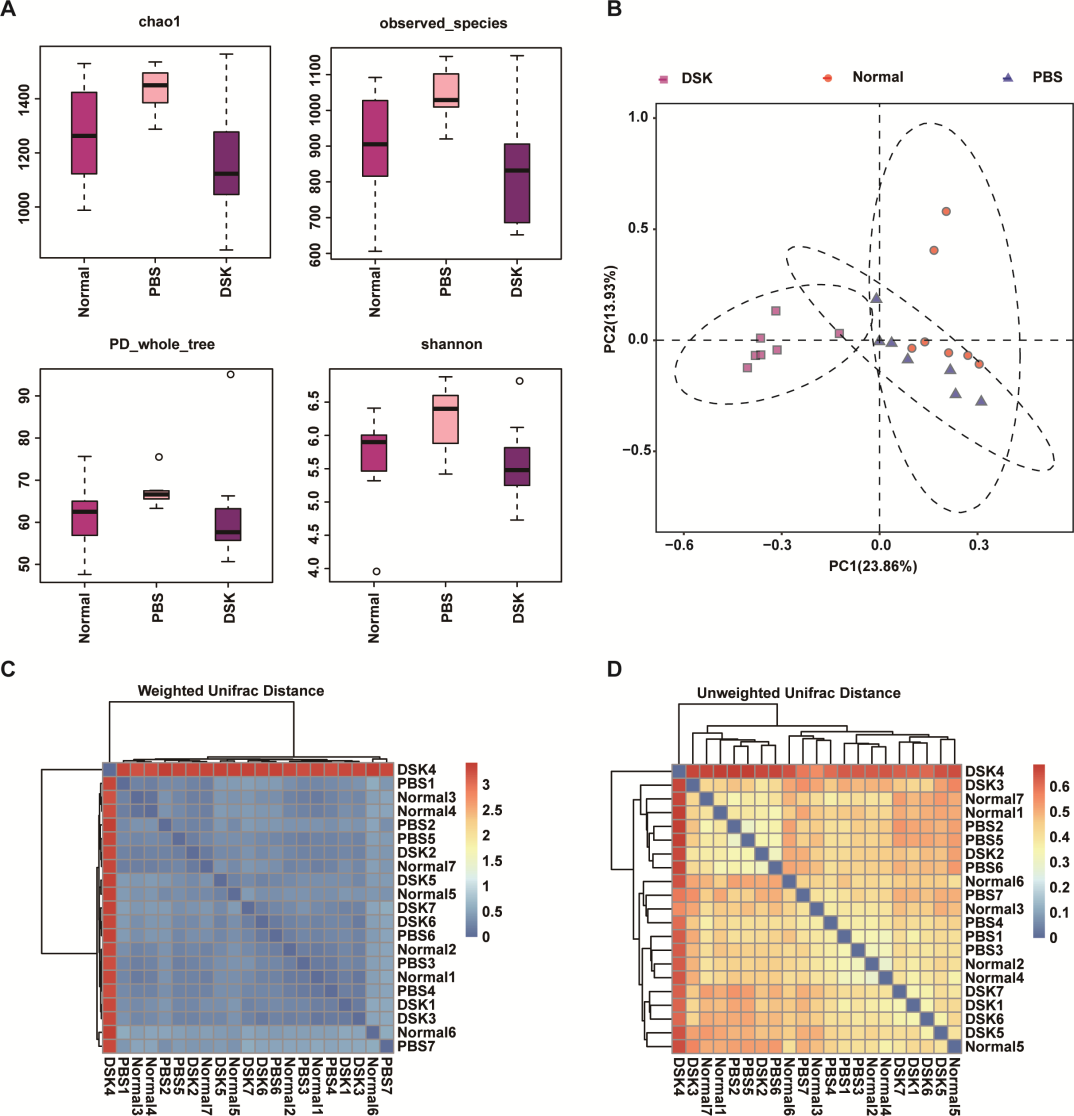
Diversity of intestinal flora in mice treated with deoxyshikonin. (A) Chao, observed species, PD whole tree, and Shannon indices reflect the α-diversity; (B) principal component analysis (PCA) to evaluate the intestinal flora β-diversity; (C) weighted analysis to evaluate intestinal microflora β-diversity; (D) unweighted analysis and evaluation of intestinal flora β-diversity.

In addition, the inhibitory effect of deoxyshikonin on 17 non-*H*. *pylori* species was examined. The MIC of deoxyshikonin against 17 non-*H*. *pylori* species, including *Escherichia coli*, exceeded 128 µg/mL, except for the MIC of 8 µg/mL against *Bacillus subtilis*, indicating that deoxyshikonin has good specificity for *H. pylori* (Table 3) and shows no effect on the intestinal microbiota (Fig. 7). Deoxyshikonin reduced the difference in gut microbiota abundance caused by *H. pylori* modeling. Moreover, the distance between the gut microbiota in each group was roughly equal, indicating that the treatment with deoxyshikonin did not increase the difference in gut microbiota.

**TABLE 3:** MIC of deoxyshikonin against different non*-H. pylori* species

Species	MIC (μg/mL)
*Lactobacillus campylosus*	> 128
*Morganella morganii*	> 128
*Enterobacter hormaechei*	> 128
*Staphylococcus haemolyticus*	> 128
*Proteus mirabilis*	> 128
*Escherichia coli*	> 128
*Staphylococcus aureus*	> 128
*Pseudomonas aeruginosa*	> 128
*Klebsiella pneumoniae*	> 128
*Acinetobacter baumannii*	> 128
*Oligotrophic bacterium maltophilia*	> 128
*Acetobacter pasteurianus*	> 128
*Saccharomyces cerevisiae*	> 128
*Bifidobacterium longum*	> 128
*Enterococcus faecalis*	> 128
*Bacillus subtilis*	8
*Campylobacter jejuni*	> 128

## DISCUSSION

The plants have antibacterial properties, but it is difficult to determine their antibacterial components (17, 18). The antibacterial activity of nine components was analyzed in purple grass. The results showed that deoxyshikonin had the best antimicrobial effect against *H. pylori*, with an MIC of 0.5 µg/mL, and was also effective against strains resistant to metronidazole, comparable to clinical drugs such as levofloxacin (19, 20). Whether a compound can be used as an ideal drug depends on their antibacterial effects and safety *in vivo* and *in vitro* (18). In our animal experimental studies, 7 mg/kg of deoxyshikonin reduced the colonization of *H. pylori* in mice, repaired the damage to gastric mucosa, and significantly decreased the release of inflammatory factors. The treatment effect *in vivo* was significantly better than that of the standard triple therapy; a large dose of deoxyshikonin (35 mg/kg) had no toxic effect on mice, thus meeting the requirements of the lead drug for safety and effectiveness. In addition, deoxyshikonin shows advantages such as good acid resistance, strong specificity, and less resistance to *H. pylori*, which can survive in the acidic environment of the stomach, whereas some antibacterial drugs are degraded by gastric acid or pepsin. This leads to a significant decrease in efficacy (21), making it necessary to combine them with antacids to achieve therapeutic effects. After co-culturing deoxyshikonin with *H. pylori* in media at different pH, deoxyshikonin exerted better antibacterial effects at a faster rate in acidic environments, exhibiting excellent acid resistance. Furthermore, this also indicated the reduced need for acid suppressants, reduced medical costs, and enhanced patient compliance. In recent years, it has been reported that the primary resistance rate of *H. pylori* to clarithromycin is 20%–50%, to levofloxacin 20%–50%, and metronidazole is 40%–70%. However, *H. pylori* may develop double, triple, or multiple resistance to these antibiotics, and the dual resistance rate of clarithromycin and metronidazole exceeds 25%. This finding implies that *H. pylori* is prone to develop resistance to these antibiotics, leading to very severe resistance. We induced the resistance of *H. pylori* to deoxyshikonin, but after 30 days of induction, no significant resistance was found, indicating that *H. pylori* is not easily resistant to deoxyshikonin, which is a rare advantage. However, the MIC of metronidazole increased 32-fold. In addition, our antibacterial spectrum and gut microbiota detection analyses found that the antibacterial spectrum of deoxyshikonin is relatively narrow and will not cause an imbalance in the intestinal microbiota, which is in agreement with the current characteristics of precise medication. Meanwhile, the drug sensitivity tests we conducted on other non-*H*. *pylori* strains are reasonable based on the differences in results caused by strains, experimental methods, and compound sources. This result indicates that deoxyshikonin has good antibacterial effects and safety *in vitro* and *in vivo* and the advantages of acid resistance, specificity, and less resistance to *H. pylori*. Therefore, it is a promising lead drug for research and development.

### Conclusion

The present study confirms that the main active ingredient found in comfrey which inhibits *H. pylori* is deoxyshikonin, which has good antibacterial effects both *in vivo* and *in vitro* with the advantages such as low toxicity, strong specificity, strong acid resistance, and difficulty in rendering *H. pylori* drug resistance. Therefore, it is an ideal, potential lead drug to alleviate the severe problem of *H. pylori* resistance.

## MATERIALS AND METHODS

### Culture and collection of standard and clinically isolated *H. pylori* strains

*H. pylori* strains (Standard 26695, NSH57, MSD132, and G27, all provided by Professor Bi Hongkai of Nanjing Medical University; *H. pylori* BS001-*H. pylori* BS016 isolated in our laboratory) and *S. aureus* were used (Table 4). *S. aureus* and other bacteria were cultured on nutrient agar plates (Baisi, BS1101) at 37°C for 1–2 days. The colonies were scraped from the plate, placed in the nutrient broth liquid medium (Baise, BS1002), and shaken for 1–2 days to obtain the *S. aureus* suspension. *H. pylori* strains were removed from the refrigerator at −80°C and centrifuged to remove the preservation solution, and thereafter, the precipitate was evenly coated on a Columbia blood agar base (OXOID, Lot 3484622) medium (OXOID, Lot 2179850) containing 10% calf serum (Pingrui, Lot 20220922) and cultured at 37°C (85% nitrogen [N_2_], 5% oxygen [O_2_], and 10% carbon dioxide [CO_2_]) for 3–4 days. The colonies were scraped from the medium, inoculated in a brain heart infusion (BHI) medium (OXOID, Lot 3555372) containing 10% calf serum, and placed under microaerobic conditions for shaking culture for 2–3 days.

**TABLE 4:** Bacterial information

Species	Origin	Medium composition
*Lactobacillus campylosus*	Guangdong Microbial Culture Collection Centre	Peptones, beef extract, yeast extract, glucose, sodium acetate, ammonium citrate dibasic, Tween-80, dipotassium hydrogenphosphate, magnesium sulfate heptahydrate, manganese sulfate heptahydrate, calcium carbonate, distilled water, and agar (no need for liquid medium)
*Morganella morganii*	Guangdong Microbial Culture Collection Center	Nutrient agar/broth
*Enterobacter hormaechei*	Clinical separation	Nutrient agar/broth
*Staphylococcus haemolyticus*	Clinical separation	Nutrient agar/broth
*Proteus mirabilis*	Clinical separation	Nutrient agar/broth
*Escherichia coli*	Clinical separation	Nutrient agar/broth
*Staphylococcus aureus*	Clinical separation	Nutrient agar/broth
*Pseudomonas aeruginosa*	Clinical separation	Nutrient agar/broth
*Klebsiella pneumoniae*	Clinical separation	Nutrient agar/broth
*Acinetobacter baumannii*	Clinical separation	Nutrient agar/broth
*Oligotrophic bacterium maltophilia*	Clinical separation	Nutrient agar/broth
*Acetobacter pasteurianus*	Guangdong Microbial Culture Collection Centre	Glucose, *Radix scutellariate*, calcium carbonate, absolute ethanol, distilled water, and agar (no need for liquid medium)
*Saccharomyces cerevisiae*	Guangdong Microbial Culture Collection Centre	Peptone, glucose, yeast extract, malt extract, distilled water, and agar
*Bifidobacterium longum*	Guangdong Microbial Culture Collection Centre	Bacto Soytone, typtone, yeast extract, glucose, saline solution, L-cysteine, 0.1% resazurin, distilled water, and agar (no need for liquid medium)
*Enterococcus faecalis*	Clinical separation	Nutrient agar/broth
*Bacillus subtilis*	Clinical separation	Sabourauds
*Campylobacter jejuni*	Clinical separation	Columbia agar base/BHI broth (with 10% serum)

### Cell culture

MGC803, BGC823 (both purchased from KeyGEN Biotech, Nanjing, China), and Ges-1 gastric epithelial cells (RRID: CVCL_EQ22) were inoculated on an RPMI medium 1640 (KeyGEN Biotech, KGM31800-500) and dosed with 10% fetal bovine serum (FBS; OriCell, FBSST-01033–500). The cells were cultured in a cell incubator containing 5% CO_2_ at 37°C.

### Determination of the MIC

The first well of a 96-well plate was added with 173.6 µL medium, then 6.4 µL shikosin (Chengdu Herbpurify Biological Co., Ltd, CAS:517–89-5), lithospermoside (Chengdu Herbpurify Biological Co., Ltd, CAS: 63492–69-3), β,β-dimethylacrylshikonin (Chengdu Herbpurify Biological Co., Ltd, CAS: 24502–79-2); β,β-dimethyl-acryl-alkannin (Chengdu Herbpurify Biological Co., Ltd, CAS:34539–65-6), lithospermic acid (Chengdu Herbpurify Biological Co., Ltd, CAS:28831–65-4), β-acetoxyisovalerylalkannin (Chengdu Herbpurify Biological Co., Ltd, CAS: 69091–17-4); isobutyrylshikonin (Chengdu Herbpurify Biological Co., Ltd, CAS:52438–12-7), acetylshikonin (Chengdu Herbpurify Biological Co., Ltd, CAS: 24502–78-1); and deoxyshikonin (Chengdu Herbpurify Biological Co., Ltd, CAS: 43043–74-9). Diluting it to the ninth well, the concentrations of the drug were 128, 64, 32, 16, 8, 4, 2, 1 and 0.5 µg/mL from the first well to the eighth, respectively. Negative well (sterile, medium, and drug only) and positive well (no drug, only medium, and bacteria) were used as the control. *H. pylori* were diluted tenfold with OD_600_ = 0.3 (1.0 × 10^8^ culture-forming units [CFU]/mL). The bacterial solution (10 µL) was distributed across the first to the ninth wells and the positive well (working concentration, 1 × 10^6^ CFU/mL) and placed under microaerobic conditions and shaking culture for 72 hours, after which the results were noted. The final working concentration of other bacteria was 1 × 10^5^ CFU/mL and shaking culture for 16–18 hours. Metronidazole was the positive control drug, and PBS was the negative control drug. An enzyme-linked immunosorbent assay was used to measure absorbance interpretation results. Each experiment was repeated three times.

### Determination of the MBC and acid resistance of deoxyshikonin

Prepare the brain–heart culture medium (pH 3.0, pH 5.0, and pH 7.0), and use the MIC detection method to double dilute so as to make the final concentration of deoxyshikonin 2, 4, and 8 µg/mL PBS as the positive control, put the 96-well plate under the micro-aerobic condition for oscillatory culture, dilute the *H. pylor*i G27 solution (100 times, 1,000 times, etc.) after a certain time of drug action (such as 2 hours), and then coat it on the Columbia agar plate, and place it in a three-gas incubator for 4–5 days. Calculate the number of bacteria growing on the agar plate. The minimum bactericidal concentration (MBC) was the drug concentration with 99.9% inhibition of bacteria.

### Antimicrobial resistance testing

*H. pylor*i G27 to the logarithmic phase, adjust the concentration of OD_600_ to 0.3 (1 × 10^8^ CFU/mL), dilute 100 times to 1 × 10^6^ CFU/mL, take 5 mL of the bacterial suspension, and add in a 50-mL sealed membrane centrifuge tube. Multiply one-fourth of the MIC concentration of deoxynivalenol by 5 mL as the dose. Check MIC every 3 days for a total of 30 days. The drug concentration was adjusted with changes in the MIC. The positive drug control group was metronidazole.

### Experimental animal modeling, detection of the amount of colonization, and pathological analyses

Thirty specific pathogen-free (SPF) C57BL/6 mice aged 6 weeks were purchased from Changsha Tianqin Biological Co., Ltd; SPF animal license number: SCXK Gui 2017–0004; animal experiment ethics number: 2019112501. Mice were administered 0.5 mL *H*. *pylori* suspension (1 × 10^9^ CFU/mL) prepared by BHI (the acclimated bacterium *H. pylori* BS001 after domestication and validation in mice using strain *H. pylori* BS001), we administered by gavage once a day, on 5 consecutive days. After 14 days of observation, the mice models were established. Thereafter, the mice were divided into the omeprazole (138.2 mg/kg) (Shanxi Jinhua Bright Star Pharmaceutical Co., Ltd, 20200901) +amoxicillin (28.5 mg/kg) (Hainan Simcere Pharmaceutical Group Co., Ltd, 02–170404) +clarithromycin (14.3 mg/kg) (Wanglintang Pharmaceutical Co., Ltd, 220601) group (the standard triple therapy group); the omeprazole (138.2 mg/kg) +deoxyshikonin (28 mg/kg) high-concentration group; the omeprazole (138.2 mg/kg) +deoxyshikonin (7 mg/kg) low-concentration group; the phosphate-buffered saline (PBS) group (mice infected with *H. pylori* but untreated); the negative control group (mice not infected with *H. pylori*). We administered medication by gavage once a day for 3 consecutive days. On the second day after drug withdrawal, the mice were euthanized, and half of the tissue from their stomach was excised. The tissue specimens were weighed, ground, diluted, coated on a Columbia plate (containing calf serum and antibiotics) under microaerobic conditions (85% N_2_, 5% O_2_, and 10% CO_2_), and cultured for 3–4 days. Thereafter, the amount of *H. pylori* colonized was calculated, with pathological analyses performed on the other half of the collected stomach tissue. This method is found in Huang’s article (22).

### Pathological safety assessment *in vivo* in mice

SPF C57BL/6 mice aged 6 weeks (the same mice as described in Section 2.4) were administered a dose of 35 mg/kg (five times the aforementioned treatment dose of 7 mg/kg) for 30 days, consecutively, and weighed. They were also weighed the day before and after gavage with tissues collected from the liver, spleen, and kidneys for hematoxylin and eosin (HE), TUNEL, IL-6, IL-1β, and TNF-α staining.

### Detection of the effect of deoxyshikonin on the morphological structure of *H. pylori* using scanning electron microscopy (SEM)

*H. pylori* G27 solution (10 mL) of cells in the logarithmic phase (1.0 × 10^8^ CFU/mL) was treated with deoxyshikonin and clarithromycin at concentrations adjusted to 4 µg/mL; incubated for 8, 12, and 24 hours, and centrifuged to remove the preservation solution. Low-speed centrifugation at 1,000 rpm prevented damage to the morphology of *H. pylori*. The resulting precipitate was fixed at 4°C overnight using the electron microscope fixative, dehydrated for 10 minutes using ethyl alcohol (30, 50, 70, 90, and 100%) respectively, dehydrated twice using 100% anhydrous ethanol, and centrifuged to obtain precipitate for freeze-drying for 2 days. The samples were coated with gold and observed by using an SEM.

### Biofilm inhibition experiments

The *H. pylori* G27 solution in the growth phase was distributed across a 96-well plate (LABSELECT, 11510), 200 µL/well at OD_600_ = 0.1, cultured under microaerobic conditions for 3 days, treated with clarithromycin and deoxyshillin both at concentrations as shown in the results, and co-incubated for 24 hours. Thereafter, the samples were treated with 10 uL Alamar blue (Solarbio A7631), and photos were taken after 4 hours. The inoculations were repeated with 0.1% crystal violet staining (McLean, CAS: 548–62-9); quantitative tests using a multifunctional enzyme marker (BioTek, America) and confocal experiments (Olympus, FV3000) using a Thermo Fisher L-7012 kit were performed on the samples. Three compound holes were established per group. Fluorescence quantification by PI/Syto9 was performed using Image J’s Split Channel feature, which splits the image to be quantified into red, green, and blue channels, and then automatically counts the fluorescence of the red and green channels, respectively.

### Effect of deoxynivalenol on the permeability of *H. pylori* cell membranes

*H. pylori* G27 in the logarithmic stage were centrifuged at 5,000 rpm for 10 minutes, collected, washed thrice using PBS, and resuspended. Thereafter, the concentration of the bacterial suspension A was adjusted to OD_600_ = 0.3 (1 × 10^8^ CFU/mL). Drug solutions and the diluted bacterial solutions were mixed according to different volumes with the final concentrations of deoxyshikonin being twice the MIC and four times the MIC, respectively, and then placed under microaerobic conditions for shaking culture for 0, 1, 2, 4, 6, and 8 hours. The light absorption values of the bacterial solutions were measured at 260 nm by using an ultraviolet spectrophotometer, with the untreated bacterial suspension used as the blank control. These procedures were repeated three times for each sample.

### Reactive oxygen species (ROS) experiments

The *H. pylori* G27 could be cultured until they reached the logarithmic stage with their concentration adjusted to 1 × 10^7^ CFU/mL, treated with 10 µM of DCFDA (Beyotime, S0033S), co-inoculated in a three-gas incubator for 1 hour, centrifuged to obtain the precipitate, and washed twice using the PBS solution. Thereafter, the samples were treated with 2, 8, and 16 µg/mL deoxyshikonin, with PBS and Rosup in the kit used as negative and positive controls, respectively. After incubation for 2 hours, the samples were observed by using a fluorescence microscope (Olympus, BX53).

### Flagella movement soft agar experiment

The standard G27 strains that had grown in BHI were collected with the concentration adjusted to OD_600_ = 3. A pipette was used to inoculate 1 µL bacterial solution into the semi-solid medium containing 0.3% agar. The medium was a drug plate containing 10% calf serum, 0.3% agar, and drugs at specified concentrations (0.125 and 0.5 µg/mL deoxyshikonin; 0.5 µg/mL amoxicillin). After 5 days, the diameters of *H. pylori* colonies growing on the medium were measured.

### The suspension drop method to observe the movement of shikonin against *H. pylori*

The logarithmic growth period G27 was taken, and the OD of the bacterial suspension was adjusted to 0.3 (1 × 10^8^ CFU/mL); a six-hole plate was used where the bacterial solution and deoxyshikonin 2 µg/mL were placed under micro-aerobic conditions and cultured for 8 hours. The bacterial solution was resuspended with PBS, and 1 µL was placed on a concave slide and observed using an ordinary optical microscope.

### Statistical analysis

The data in the bar chart were expressed as mean standard error (SEM: standard error of mean). All statistical analyses were performed using Graphpad PRISM software, Version 7.0. All data were subjected to Student’s *t*-tests and single-factor analysis of variance (ANOVA).
