# Erratum for Xu et al., “Deoxyshikonin: a promising lead drug grass against drug resistance or sensitivity to *Helicobacter pylori* in an acidic environment”

**DOI:** 10.1128/aac.01840-24

**Published:** 2025-01-27

**Authors:** Jia-yin Xu, Hui-hua Dong, Li-juan Liao, Shi-xian Yang, Lu-yao Wang, Hao Chen, Peipei Luo, Liang Huang, Ai-xing Guan, Yan-Qiang Huang

## ERRATUM

Volume 68, no. 10, e00959-24, 2024, https://doi.org/10.1128/aac.00959-24. Page 14: Affiliation 6 should read “Guangxi Clinical Medical Research Center for Hepatobiliary Diseases, Baise, China.”

